# Monocyte Distribution Width for Sepsis Diagnosis in the Emergency Department and Intensive Care Unit: A Systematic Review and Meta-Analysis

**DOI:** 10.3390/ijms26157444

**Published:** 2025-08-01

**Authors:** Jessica Elisabetta Esposito, Milena D’Amato, Giustino Parruti, Ennio Polilli

**Affiliations:** 1 Department of Innovative Technology in Medicine and Dentistry, University “G. D’Annunzio”, 65122 Chieti, Italy; 2 Department of Biotechnological and Applied Clinical Sciences, University of L’Aquila, 67100 L’Aquila, Italy; milena.damato@asl.pe.it; 3 Infectious Diseases Unit, Pescara General Hospital, 65124 Pescara, Italy; giustino.parruti@asl.pe.it; 4 Clinical Pathology Unit, Pescara General Hospital, 65124 Pescara, Italy

**Keywords:** MDW, sepsis, ED, ICU, early diagnosis, meta-analysis

## Abstract

We planned a systemic review and meta-analysis to evaluate the diagnostic accuracy of Monocyte Distribution Width (MDW) in aiding the diagnosis of sepsis in the Emergency Department (ED) and Intensive Care Unit (ICU). A systematic literature search was performed in PubMed, Scopus, and OVID to retrieve studies published up to 29 January 2024. We examined results using mean difference and conducted a diagnostic test accuracy (DTA) meta-analysis using a bivariate random effects model. Pooled results showed that MDW was significantly higher in sepsis patients admitted to the ED (MD = 5.59, 95%CI: 4.14–7.05) or to the ICU (MD = 8.30, 95%CI: 2.98–13.62). Nine studies conducted in the ED were included in the DTA review. The overall sensitivity was 0.80 (95%CI: 0.75–0.85), the specificity was 0.76 (95%CI: 0.66–0.83), and the false-positive rate (FPR) was 0.24 (95%CI: 0.17–0.34). Three studies were conducted in the ICU, but only two were included in the DTA meta-analysis. Of the 662 patients admitted to the ICU, 175 developed sepsis, showing higher MDW values than non-septic patients. However, significant heterogeneity was noted among the studies. MDW is a helpful biomarker for sepsis in adult patients admitted to the ED and ICU. In the ED, MDW could aid clinicians in ruling out sepsis.

## 1. Introduction

Sepsis is a life-threatening illness and one of the leading causes of death worldwide [1,2,3,4]. Patients may be diagnosed with sepsis upon admission to the emergency department (ED), or they may develop it during their hospital stay, such as in the intensive care unit (ICU), due to invasive procedures, prolonged stays, exposure to antibiotics, or severe illness [5,6,7,8]. New diagnostic systems or accurate laboratory biomarkers are needed to improve the early identification of patients with infection at high risk of developing organ dysfunction [9,10].

Over 200 biomarkers are currently being evaluated for diagnosing sepsis and stratifying patients with infection [11]. Recently, Monocyte Distribution Width (MDW) has been introduced as a novel biomarker for aiding in the recognition of sepsis. It represents the standard deviation of the monocyte population volume and is determined using volume, conductivity, and scatter (VCS) technology [12].

Monocytes are cells of the innate immune system that play a key role in the first line of defense against infection. Other cells involved in this phase include neutrophils, eosinophils, basophils, γδ T cells, and natural killer cells. This response is established within the first hours or days following microbial invasion and hinders the spread of most pathogens. Monocytes recognize pathogen-associated molecular patterns (PAMPs) and damage-associated molecular patterns (DAMPs) through pattern recognition receptors (PRRs); this triggers intracellular signaling pathways and activates the production of proinflammatory cytokines, such as IL-1β, IL-18, TNFα, and IL-6, as well as chemokines [13]. These processes lead to changes in monocyte morphology, such as increased volume and filopodia formation. VCS technology indirectly mirrors these morphological changes in immature and reactive cells by mimicking the microscopic evaluation of a peripheral blood smear. VCS technology measures cell volume using electrical impedance. Cells pass through a small aperture where a current is applied by two submerged electrodes positioned on either side. As each cell passes through this area, the impedance of the electrical path is measured, producing an electrical pulse. MDW is calculated as the standard deviation in the volume of the peripheral monocytes [14]. In the early phase of sepsis, MDW may increase in response to proinflammatory stimuli, reflecting changes in the morphology and function of circulating monocytes and reproducing the features of a highly heterogeneous cell population [15,16].

In April 2019, the Food and Drug Administration (FDA) approved MDW as a sepsis biomarker, and a value of 20.0 is indicated as the cut-off beyond which the suspicion of sepsis should be raised for adult patients admitted to the ED [17].

Several reviews have investigated the use of MDW in diagnosing sepsis [16,18,19,20,21,22]. Many of these studies were conducted with different objectives. For instance, Malinowska et al. (2023) examined MDW in various conditions, including sepsis, infections, and SARS-CoV-2 [19]. In contrast, studies by Huang et al. (2023) [20] and Motawea et al. (2023) [21] aimed to compare MDW with other infection biomarkers, such as Procalcitonin (PCT) and C-reactive protein (CRP). Finally, Eisinger et al. (2025) investigated the use of MDW for diagnosing sepsis by conducting two separate analyses based on sepsis diagnostic criteria [22].

Here, we systematically reviewed the published studies on the performance of MDW in aiding the diagnosis of sepsis in the ED and ICU.

## 2. Materials and Methods

The study protocol was registered with the International Prospective Register of Systematic Reviews (CRD42024512930).

### 2.1. Study Selection, Data Extraction, and Quality Assessment

We performed a systematic literature search on 29 January 2024, in PubMed, Scopus, and OVID to identify all relevant studies for our review. We did not apply any time filter to the literature search; thus, each study retrieved by running the keywords (Table S1) after duplicate removal was considered suitable for the screening. For the purpose of our review, we included any randomized controlled trials and prospective, retrospective, and observational studies (such as cohort studies, case-control studies, or cross-sectional studies) that examined the relationship between MDW and sepsis diagnosis among adult patients admitted to the ICU and the ED. We only included articles published in English. Duplicate results were considered only once. We excluded abstracts, posters, case reports, editorials, commentaries, reviews, meta-analyses, protocols for reviews, and guidelines. We also excluded articles with inappropriate study designs such as surveys, in vitro and in vivo studies, studies without a clear sepsis group identified, and studies enrolling non-consecutive patients. Additionally, we excluded studies involving pediatric patients (<18 y), patients admitted to wards other than the ICU and ED, and patients with COVID-19 because they investigated inappropriate populations for this study. We also excluded pregnant women, because monocyte and adaptive immune system activation generally occur during pregnancy and may influence MDW results. Articles that were deemed out of topic for the purpose of this review, as well as articles that reported different outcomes or incomplete data, were excluded. The search strategies and terms, as well as the inclusion and exclusion criteria, are summarized in the Supplementary Materials, Table S1.

After importing the initial search results from three databases into Zotero (Corporation for Digital Scholarship, Vienna, VA, USA) [23], a free and open-source reference management software, duplicates were removed. Retrieved results were then imported into Rayyan (Rayyan Systems Inc., Cambridge, MA, USA) [24], a screening tool for systematic review. Based on the eligible criteria, two reviewers (J.E.E. and M.D’A.) independently screened titles and abstracts and excluded irrelevant searches. The discrepancies between the two investigators were resolved by consulting with another senior investigator (E.P.). The full text of the remaining searches was retrieved and examined to include articles.

The selected studies went through the data extraction process. Data extraction was independently performed by J.E.E. and E.P. The following information from the included studies were collected: features of the study (study design, authors, years of publication, setting and diagnostic criteria), population (sample size, gender, and mean or median age), and investigated outcomes (mean or median values of MDW in patients and controls, cut-offs of MDW). The investigators also recorded data on prevalence, true-positive, false-positive, true-negative, and false-negative values, as well as the sensitivity and specificity of MDW. Additionally, the type of anticoagulant used for storing blood samples was noted. The supplementary files of the included studies were also examined for data extraction. When the mean and standard deviation of MDW could not have been directly retrieved from a study, they were estimated using median, interquartile range, and sample size [25,26]. Where necessary, we combined the numbers, means, and standard deviations for different groups into a single group, using formulas recommended by the Cochrane methodology [26,27].

The Quality Assessment of Diagnostic Accuracy Studies 2 (QUADAS-2) tool [28] was used to evaluate the quality of diagnostic test accuracy studies by using four domains of bias and applicability: patient selection, index test, reference standard, and flow and timing. The risk of bias of each included study was judged as low, high, or unclear by two independent reviewers (J.E.E. and E.P.). Discrepancies were extensively discussed until a consensus was reached.

### 2.2. Statistical Analysis

Meta-analyses were performed by E.P. Mean and standard deviation of MDW were pooled for continuous data. The mean difference (MD) with 95% confidence interval (95%CI) among the study groups was calculated, and the data was displayed using Forest plot analysis. We further performed predefined subgroup analyses to examine the heterogeneity among studies stratified by diagnostic criteria (SEPSIS-2 or SEPSIS-3 criteria), sepsis prevalence (≥7% or <7%), sample size (≥1320 or <1320), published MDW cut-off value in different studies (≥21 U or <21 U), and anticoagulant (dipotassium ethylenediaminetetra-acetic acid, K2-EDTA or tripotassium ethylenediaminetetra-acetic acid, K3-EDTA). Several factors may influence biomarker accuracy, including disease prevalence and the characteristics of the study. For instance, increased prevalence values could increase or decrease sensitivity and/or specificity [29]. Similarly, the sample size may influence heterogeneity, with smaller samples tending to show higher levels of heterogeneity than those with larger enrolments [30]. Generally, the larger the sample size, the greater the certainty of the accuracy of the estimates of sensitivity and specificity [31].

We also considered additional variables reported to affect MDW accuracy and increase heterogeneity among studies. These include the cut-off value, the use of SEPSIS-3 or SEPSIS-2 criteria, and the type of coagulant used. EDTA may cause erythrocyte contraction, affecting mean corpuscular and platelet volumes. These effects are more pronounced with K3-EDTA than with K2-EDTA. Therefore, samples collected in K2-EDTA tubes report MDW values that are systematically two points lower than those collected in K3-EDTA tubes. Thus, MDW values should be interpreted based on the specific type of EDTA tube used. As described by Wang et al. (2021), we used data-driven approaches to dichotomize sample size, sepsis prevalence, and published cut-off of MDW [32], thus selecting the second quartile value of these variables and rounding it to the nearest whole number [33]. Only three studies involving ICU patients were available for our analysis. Due to the small sample sizes, which may not yield significant or generalizable results, we did not conduct a subgroup analysis.

We employed meta-regression using Meta-Disk 2.0 [34] to investigate significant factors contributing to heterogeneity. When appropriate, we assessed heterogeneity using the I^2^ statistic. According to the Cochrane manual, an I^2^ statistic greater than 50% indicates heterogeneity [35,36].

We also performed a diagnostic meta-analysis using the methods described in Chapter 10 of the Cochrane Handbook [37]. According to these criteria, we statistically calculated sensitivities, specificities, likelihood ratios (LR), and diagnostic odds ratios (DOR) using a bivariate random effects model [38,39,40].

We considered MDW positive if its value was above the threshold defined in the primary study, and negative if it was below this threshold. Cross-classifying these results with the reference standard generated the numbers of true positives, false positives, true negatives, and false negatives for each study. We analyzed data from the two-by-two tables and calculated sensitivity and specificity for each study. The two-dimensional nature of the diagnostic data was preserved by analyzing the logit-transformed sensitivity and specificity of each study in a single model, taking into account both within-study and between-study variability. We present individual study results by plotting the estimates of sensitivity and specificity, and their 95%CI, in receiver operating characteristic (ROC) scatter plots. We estimated the sample size-weighted aggregate prevalence of the disease, which we then used to calculate the summary of the negative and positive predictive values (NPV and PPV), as described by Leeflang et al. (2012) [41]. We analyzed heterogeneity by calculating the variances of logit sensitivity and specificity, the area of the 95% prediction ellipse, and the median odds ratio (OR) [42].

All meta-analyses were performed with R version 4.1.1 (2021-08-10, Boston, MA, USA) [43] using the “meta” package, Meta-disk 2.0 [34], and STATA 14 using the “metandi” package [44].

## 3. Results

**Figure 1:**
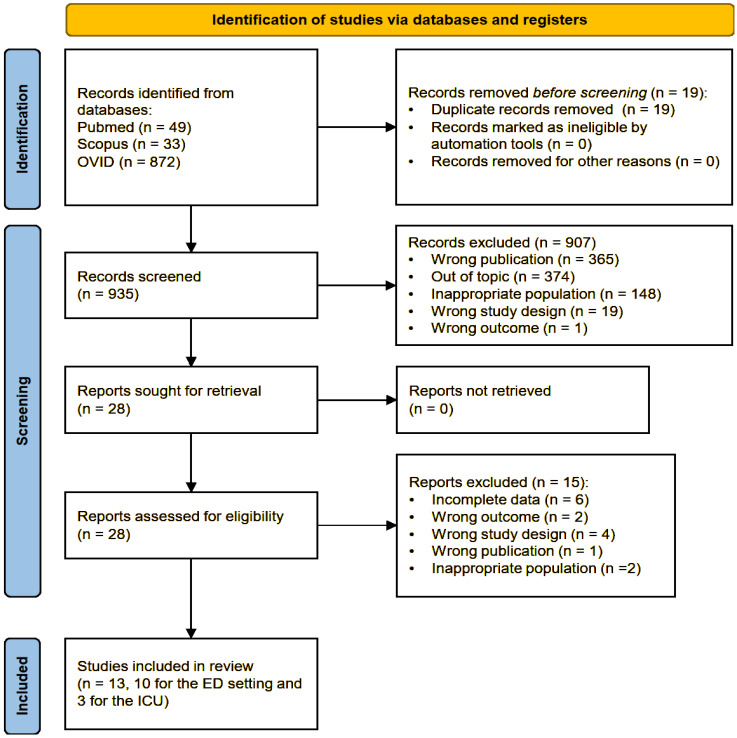
PRISMA flow diagram of the study selection process.

The literature search across three databases yielded an overall result of 954 total publications (49 from PubMed, 33 from Scopus, and 872 from OVID). After removing all duplicates, 935 articles were screened by title and abstract. Nine hundred seven records were excluded as they were deemed out of topic for the purpose of this review (374), belonged to a wrong type of publication (365), investigated an inappropriate population (148) or outcome (1), or used an inappropriate study design (19). Twenty-eight articles were sought for retrieval and assessed for eligibility, among which 15 were excluded. Thirteen studies were finally included in this systematic review, of which 10 were conducted enrolling ED populations and 3 were conducted on ICU patients. The study selection process is schematically summarized in the Preferred Reporting Items for Systematic reviews and Meta-Analyses (PRISMA) flow-chart (Figure 1) [45].

**Figure 2:**
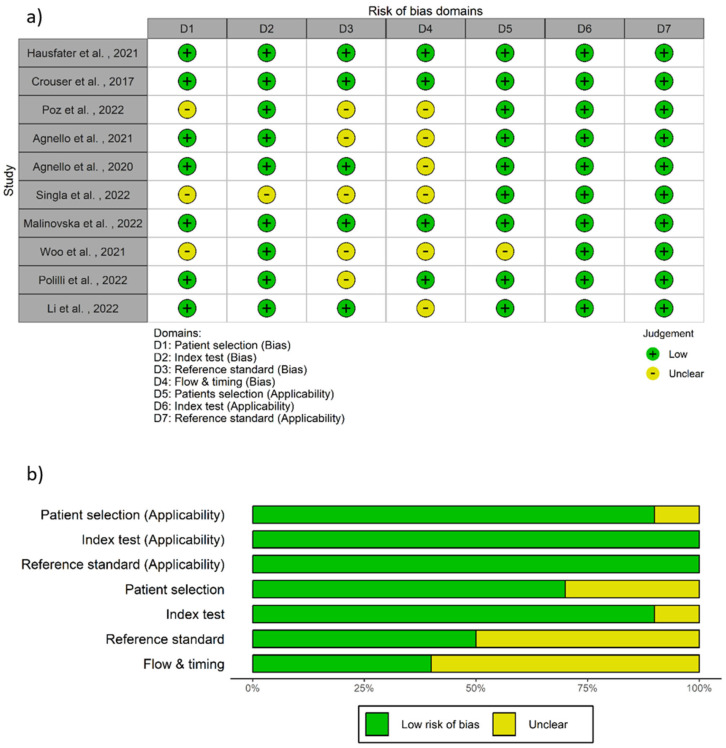
The risk of bias and applicability concerns graph. Quality assessment for the included studies enrolling the ED population (**a**) presented as percentage (**b**) [15,50,51,52,53,54,55,56,57,58]. (Quality Assessment of Diagnostic Accuracy Studies, QUADAS–2).

Several studies have evaluated the diagnostic ability of MDW to early identify sepsis [14,15,46,47,48,49,50,51,52,53,54,55,56,57,58]. Ten included studies were conducted in ED populations (Table S2) [15,50,51,52,53,54,55,56,57,58]; of these, one [55] resulted at increased risk of bias from our quality assessment (Figure 2a).

The results of the quality assessment according to the QUADAS-2 criteria are shown in Figure 2 In the domain of patient selection (D1, Figure 2a), three studies (30%) received an unclear risk of bias judgment due to an insufficient description of the enrollment method [52,55,57]. In the index test domain (D2, Figure 2a), one study (10%) was unclear because it did not sufficiently describe whether the index test results were interpreted without knowledge of the reference standard results [55]. In the reference standard domain (D3, Figure 2a), we judged five studies (50%) to have an unclear risk of bias because the authors did not explicitly state whether their reference standard results were interpreted with knowledge of the index test results [15,52,54,55,57]. For flow and timing (D4, Figure 2a), six studies (60%) had an unclear risk of bias. Five studies were judged unclear because we did not retrieve enough information from these articles to judge if the intervals between the index test and the reference standard were appropriate [15,52,53,57,58]. One study did not clearly indicate whether all participants underwent the same reference standard [55]. As for the applicability, one study had an unclear applicability in the patient selection domain (D5, Figure 2a) as it enrolled only patients with suspected infection [57], whereas all other studies were at a low risk.

From our risk-of-bias assessment, the study by Singla et al. (2022) [55] had an increased risk of bias compared to the other included articles. Since the meta-analysis result could be affected by biases of the included studies, we decided to mitigate the potential bias in the research by excluding this article from the analysis [59]. Finally, nine studies conducted on ED populations were included in the meta-analysis [15,50,51,52,53,54,56,57,58].

## 4. Meta-Analyses of MDW Values

**Figure 3:**
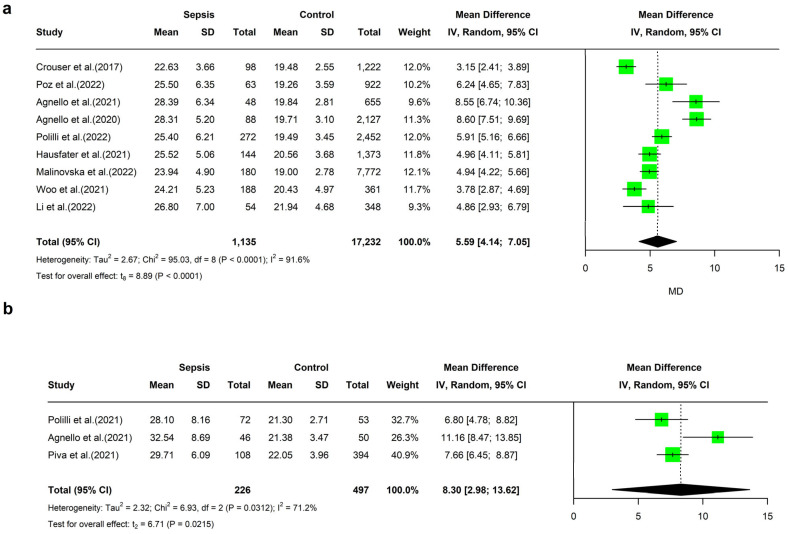
Forest plot of mean difference and 95% confidence interval of MDW values in sepsis and non-septic patients admitted in ED (**a**) [15,50,51,52,53,54,56,57,58] and in ICU (**b**) [14,48,49].

MDW values in the sepsis groups were significantly higher compared with the non-septic group of patients. Therefore, the overall pooled MD in the studies enrolling patients in the ED was 5.59 (95%CI: 4.14–7.05, I^2^ = 92%) (Figure 3a).

In the subgroup analysis, significant *p*-values were estimated for MDW cut-offs higher than 21 (6.91, 95%CI: 3.96–9.85 vs. 4.46, 95%CI: 3.00–5.92, *p* = 0.02), for the use of K3-EDTA instead of K2-EDTA as anticoagulant (6.91, 95%CI: 4.02–6.47 vs. 4.40, 95%CI: 2.34–6.47, *p* = 0.03), and for studies with a sepsis prevalence more than 7% (4.51, 95%CI: 3.12–5.90 vs. 7.03, 95%CI: 4.13–9.94, *p* = 0.01, Table S3).

Three studies included patients hospitalized in the ICU (Table S4) [14,48,49]. When compared with the non-septic group, sepsis was associated with higher MDW values, with a pooled mean difference between groups of 8.30, 95%CI: 2.98–13.62 (*p* = 0.02, Figure 3b). However, as in the ED setting, we also found a significantly high level of heterogeneity in the ICU setting (I^2^: 71.2%).

## 5. Diagnostic Meta-Analysis

The main results of our analysis considering the diagnostic performance of MDW for sepsis are shown in Table 1.

We considered nine studies conducted in emergency departments, which included a total of 18,367. Among these, 1135 were found to be septic, resulting in a prevalence of 6%.

The overall sensitivity and specificity of MDW were 0.80 (95%CI: 0.75–0.85) and 0.76 (95%CI: 0.66–0.83), respectively, with a false-positive rate (FPR) of 0.24 (95%CI: 0.17–0.34). Five studies set a threshold of MDW at approximately 20 [51,52,56,57,58], two set it close to 23 [15,53], and two other studies published further different thresholds: 22 [54] and 21.5 [50]. Sepsis was defined using the SEPSIS-2 criteria in five studies [15,51,52,53,54], and the SEPSIS-3 criteria in four studies [50,56,57,58]. The sample-size-weighted aggregate disease prevalence was 9% for studies conducted in the ED. The pooled NPV and PPV were 0.97 and 0.24, respectively.

**Figure 4:**
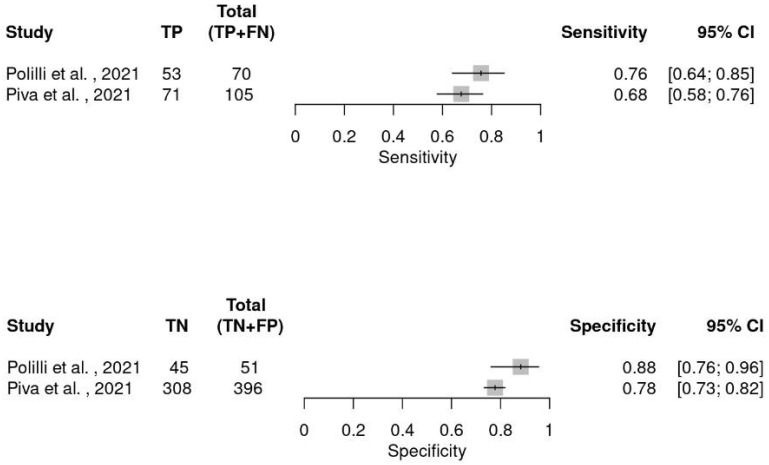
Forest plot of sensitivity and specificity of MDW across all included studies conducted in ICU [14,48].

Only two studies conducted in ICUs had sufficient data to perform a diagnostic meta-analysis [14,48]. Six hundred and sixty-two patients, of whom 175 were septic, were included in the final analysis, and the sensitivity and specificity are shown in Figure 4.

## 6. Investigation of Heterogeneity

**Figure 5:**
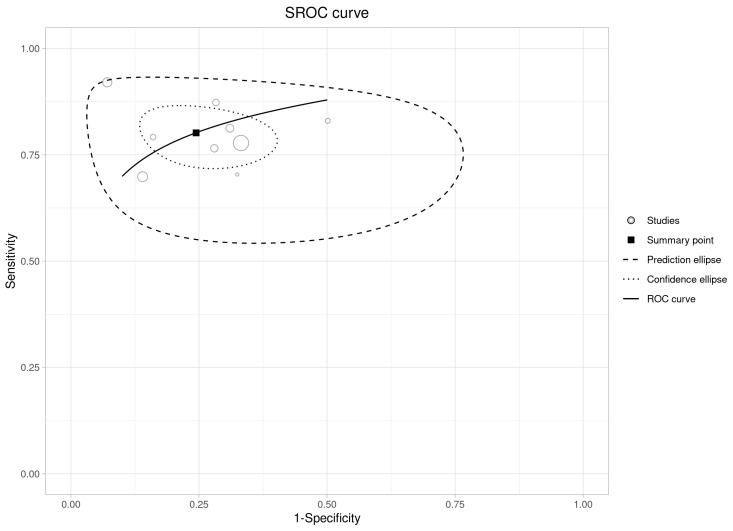
The SROC curve of the bivariate model showing the accuracy of MDW.

For meta-analysis enrolling patients in the ED, the variances of logit sensitivity and logit specificity were 0.137 and 0.508, respectively. The odds of being “true positive’’ are 1.423 times more likely (median) when a diseased individual is switched from a lower sensitivity study to a higher sensitivity study [42]. The median OR for specificity is 1.974, indicating that the odds of a true-negative result are almost two times more likely (median) when a non-diseased individual is switched from a study with lower specificity to a higher specificity study. The area of the 95% prediction ellipse is 0.236, and the I^2^ _Biv_ is 0.92. Taking all these results into account, we can conclude that there is high heterogeneity [42] (Figure 5 and Table S5).

## 7. Subgroup Analysis and Meta Regression

### 7.1. Diagnostic Criteria

Table 2 and Table 3 show subgroup analysis and meta-regression, including sensitivity, specificity, and false-positive rates. The summary sensitivity and specificity for sepsis prediction using the SEPSIS-2 criteria were 0.81 (95%CI: 0.74–0.87) and 0.83 (95%CI: 0.76–0.88), respectively, with a false-positive rate of 0.17 (95%CI: 0.12–0.24). The summary sensitivity and specificity for sepsis prediction using the SEPSIS-3 criteria were 0.79 (95%CI: 0.71–0.85) and 0.64 (95%CI: 0.52–0.74), respectively, with a false-positive rate of 0.36 (95%CI: 0.26–0.48). There was no significant change in relative sensitivity between the two groups (*p* = 0.684), but a significant difference in specificity was observed (*p* = 0.012).

### 7.2. Cut Point of MDW

Four studies used a cut-off value of MDW ≥21 to diagnose sepsis [15,50,53,54]. Summary estimates of sensitivity and specificity were 0.81 (95%CI: 0.73–0.87) and 0.85 (95%CI: 0.77–0.9), respectively, with a false-positive rate of 0.15 (95%CI: 0.1–0.23). Five studies used a cut-off of MDW <21 [51,52,56,57,58]; the summary estimates of sensitivity and specificity for this group of studies were 0.8 (95%CI: 0.73–0.85) and 0.66 (95%CI: 0.56–0.75), respectively, with a false-positive rate of 0.34 (95%CI: 0.25–0.44). The relative sensitivity did not change significantly (*p* = 0.80), but the relative specificity was significantly different (*p* = 0.009, Table 2 and Table 3).

### 7.3. Sepsis Prevalence

Four studies reported a sepsis prevalence value <7% [15,52,53,56]. Summary estimates of sensitivity and specificity were 0.84 (95%CI: 0.78–0.89) and 0.81 (95%CI: 0.69–0.89), respectively, with a false-positive rate of 0.19 (95%CI: 0.11–0.30). Five studies reported a sepsis prevalence ≥7% [50,51,54,57,58]; summary estimates of sensitivity and specificity for this group of studies were 0.77 (95%CI: 0.71–0.82) and 0.7 (95%CI: 0.57–0.82), respectively, with a false-positive rate of 0.3 (95%CI: 0.19–0.43). We found no significant differences in relative sensitivity (*p* = 0.084) or relative specificity (*p* = 0.191) between the two groups (Table 2 and Table 3).

### 7.4. Sample Size

Four studies reported a sample size of <1320 patients [15,52,57,58]. Summary estimates of sensitivity and specificity were 0.81 (95%CI: 0.73–0.87) and 0.7 (95%CI: 0.54–0.82), respectively, with a false-positive rate of 0.3 (95%CI: 0.19–0.46). Five studies reported a sample size of ≥1320 patients [50,51,53,54,56]; summary estimates of sensitivity and specificity for this group of studies were 0.8 (95%CI: 0.73–0.85) and 0.8 (95%CI: 0.69–0.88), respectively, with a false-positive rate of 0.2 (95%CI: 0.13–0.31). We found no significant differences in relative sensitivity (*p* = 0.759) or relative specificity (*p* = 0.249) between the two groups (Table 2 and Table 3).

### 7.5. Anticoagulants

Four studies collected blood samples using K2-EDTA as anticoagulant [51,52,56,57]. Summary estimates of sensitivity and specificity were 0.81 (95%CI: 0.74–0.87) and 0.66 (95%CI: 0.53–0.76), respectively, with a false-positive rate of 0.34 (0.24–0.47). Four studies collected the blood samples using a K3-EDTA anticoagulant [15,50,53,54]; summary estimates of sensitivity and specificity for this group of studies were 0.81 (95%CI: 0.73–0.87) and 0.85 (95%CI: 0.77–0.9), respectively, with a false-positive rate of 0.15 (95%CI: 0.1–0.23). We found no significant differences in relative sensitivity (*p* = 0.933), but relative specificity was significantly different between the two groups (*p* = 0.017). A study [58] was not considered in this subgroup analysis since we could not retrieve the information about the anticoagulant used to store blood samples (Table 2 and Table 3).

## 8. Publication Bias

**Figure 6:**
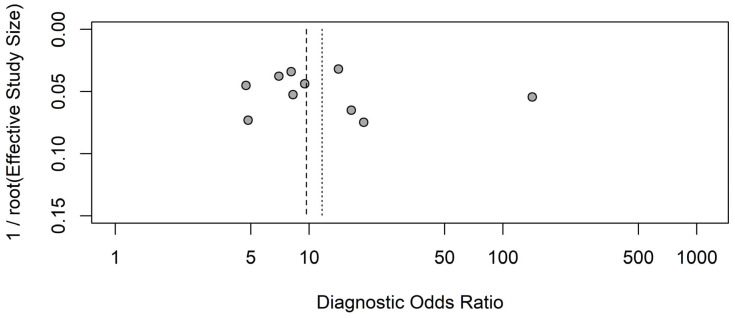
Deeks’ funnel plot asymmetry test for publication bias. The vertical lines show the pooled common effects model (dashed line) and the pooled random effects model (dotted line).

In the evaluation of publication bias, the result showed that there was no publication bias (bias = 13.18; SE = 22.27; *p* = 0.57). The results of Deek’s funnel plot are shown in Figure 6.

## 9. Discussion

MDW was introduced as a sepsis indicator only a few years ago. Since then, many specialists, including clinical pathologists and physicians, have wondered how to use it in their clinical experience. Functions and characteristics of biomarkers can vary across settings, and their role in emergency departments and intensive care units may change. In the ED, sepsis biomarkers may help identify patients with sepsis and those without infection, who can safely avoid antibiotics. In the ICU, in combination with other clinical and laboratory examinations, MDW may be used to monitor patients for early-onset infections (e.g., nosocomial infections) and sepsis progression (e.g., assisting with decisions related to the continuation, modification, or de-escalation of antimicrobial therapy) [60].

The main results of the meta-analysis involving studies in ED are shown in Table 1.

Patients with sepsis had higher mean values of MDW than the control group (Figure 3a). Diagnostic meta-analysis confirmed these findings. Therefore, while the overall sensitivity was high, the specificity was slightly lower, resulting in a very low false-positive rate. The results did not change when a study enrolling only patients at increased risk of infection was excluded [58]. These findings are in line with previous reports and further confirm that MDW is associated with moderate-to-higher sensitivity in diagnosing sepsis in the ED setting. When used as a standalone test to screen for sepsis in the ED, it is associated with a false-negative rate of approximately 1%, indicating its propensity to rule out disease. These results were confirmed by a high summary negative predictive value and a low positive predictive value. However, as the MDW results did not perfectly match negative cases, its value should be interpreted considering all other clinical and laboratory findings.

MDW is an indicator of changes in peripheral monocyte volume. As monocytes play a key role in the early stages of the adaptive immune response, changes in the MDW value may occur during the initial phases of infection. A simple blood count can quickly help clinicians identify sepsis, making it particularly useful in the ED setting [61]. As a result, many studies have been conducted to improve the early recognition of sepsis in the ED.

Patients admitted to the ICU are at risk of infection, and this risk may increase with the number of days spent in the ward [62]. Therefore, in this setting, patients are monitored daily for infection, and several methods have been proposed to stratify this risk [63]. The latest sepsis management guidelines (SEPSIS-3) recommend routine monitoring of the Sequential Organ Failure Assessment (SOFA) score. Many biomarkers have also been proposed for this purpose. Among them, PCT is the most widely accepted in the ICU. Daily or twice-daily measurements have improved sensitivity and facilitated de-escalation of antibiotics. However, no biomarker has yet demonstrated optimal performance to be accepted as a reference standard for the diagnosis of sepsis. MDW is measured using a simple Blood Cell Count analyzer, and its results are rapidly available [14,48]. Therefore, daily MDW measurements in the ICU are the object of emerging investigations, and their increase during hospitalization can be used as an early warning of the onset of infection [14,48]. However, studies conducted in the ICU are even more limited, and more research is needed to determine the accurate role of MDW in sepsis diagnosis in this setting.

Subgroup and meta-regression analyses were conducted to identify the sources of heterogeneity. The SEPSIS-2 criteria and the use of K3-EDTA enhanced the specificity of MDW, with both factors significantly affecting the heterogeneity. The varying MDW cut-off values employed in different studies also contributed to the observed heterogeneity.

## 10. Strengths and Weaknesses of the Review

We performed a subgroup analysis taking into account the sample size, the kind of anticoagulant used in the MDW analysis, sepsis prevalence, and sepsis definitions. The bivariate analyses supported the finding that the studies using SEPSIS-2 as screening sepsis criteria had a higher pooled mean difference in MDW than those using SEPSIS-3 criteria. Specifically, compared to research employing the SEPSIS-2 criteria, a subgroup of studies using the SEPSIS-3 criteria showed a significantly lower relative specificity with a higher FPR of MDW. Over the years, various criteria have been introduced to help physicians detect sepsis early. The Systemic Inflammatory Response Syndrome (SIRS) criteria, established in 1991, turned out to be not specific for sepsis detection in the case of patients with pancreatitis, pulmonary embolism, acute coronary syndrome, and other non-infectious diseases [64]. In 2016, a newly revised definition based on SEPSIS-3 criteria was introduced in clinical experience, assigning the use of the qSOFA score to non-ICU settings [65]. Nevertheless, the qSOFA score demonstrated optimal specificity but lower sensitivity than SIRS-2, making it unhelpful as a screening tool for “ruling out” sepsis in its early phase [66]. Therefore, there is no preferred method for detecting sepsis in its early phases in the ED. Although early detection of sepsis during clinical experience primarily depends on clinical judgment, both the SEPSIS-2 and SEPSIS-3 criteria for sepsis diagnosis can be utilized as diagnostic support for sepsis detection in the ED. Consequently, while many clinicians and researchers in real clinical practice have adopted the latest SEPSIS-3 criteria for identifying sepsis, others still use the previously published SEPSIS-2 criteria [67]. Consequently, several studies have used the SEPSIS-2 instead of the SEPSIS-3 criteria as the reference standard for sepsis [15,50,51,52,53,54,67]. Thus, as in previously published meta-analyses [20,22], we included sepsis criteria in our subgroup analysis.

Specific experimental conditions may influence MDW results, such as, for instance, the use of K3-EDTA as the preferred anticoagulant in the analysis tube rather than K2-EDTA [68]. In our subgroup analysis, the pooled mean difference from studies using K3-EDTA was higher than studies using K2-EDTA. Additionally, the bivariate analysis confirmed this finding and showed that K3-EDTA was significantly associated with better specificity and lower FPR.

The MDW cut-off estimates showed heterogeneous findings across studies. As a result, in the subgroup analysis, we observed variations in specificity values. Studies with a cut point >21 had better specificity and a >50% reduction in FPR, while sensitivity remained unchanged.

## 11. Agreement and Disagreement with Other Studies

To date, the number of published diagnostic meta-analyses investigating the role of MDW in sepsis diagnosis is still low [18,19,20,21,22]; of these, only three meta-analyses [19,20,22] have assessed the risk of bias for each included study. In accordance with our study, the risk of bias assessment performed by Huang et al. (2023) [20] did not identify any study at high risk of bias in any domain. Instead, the risk assessment performed by Malinovska et al. (2023) [19] and Eisinger et al. (2025) [22] highlighted a few studies with a high risk of bias in at least one domain. The difference with our risk of bias assessment could be explained by considering the more stringent criteria applied by those authors for the judgment of each domain.

As mentioned in the Introduction Section, our objectives and methodologies differ from those reported in other meta-analyses. Agnello et al. (2022), including a total of 9475 individuals, showed that MDW had good diagnostic accuracy for early detection of patients with sepsis [18], but they did not perform a subgroup analysis. Malinovska et al. (2023) included studies evaluating MDW performance in several infectious diseases, including sepsis, COVID-19, influenza, and malaria [19]. Huang et al. (2023) [20] and Motawea et al. (2023) [21] conducted a systematic review to compare the effectiveness of MDW with other established biomarkers, such as PCT and CRP, in predicting sepsis. Recently, Eisinger et al. (2025) published a large meta-analysis investigating the different performances in sensitivity and specificity of MDW for different sepsis definitions using the bivariate mixed-effects model method [22].

In 2017, Crouser et al. published the first study correlating MDW with early sepsis diagnosis in the ED [51]. Since then, many studies investigating MDW have been conducted in the ED and ICU. In 2019, the US FDA approved MDW as an additional test for diagnosing sepsis in adult patients in the ED [17]. Thus, unlike previous investigations that included all studies from different clinical settings in their meta-analyses, we analyzed studies admitting patients in the ED and ICU separately [18,19,20,21].

Additionally, we used a preregistered protocol, a rigorous methodology in line with the Cochrane guidelines, and a validated reporting checklist. In line with current recommendations, we statistically pooled sensitivities, specificities, likelihood ratios, and diagnostic odds ratios using a bivariate random effects model. We assessed heterogeneity using the area of the 95% prediction ellipse and the median odds ratio [34] and explored potential sources of heterogeneity through subgroup and meta-regression analyses.

Our analysis confirmed the main findings of previously published studies [18,19,20,22]. Similar to our results, two comprehensive systematic reviews by Huang et al. (2023) and Eisinger et al. (2025), which included large patient samples, reported that the pooled specificity could be significantly lower in studies using the SEPSIS-3 criteria than in studies using the SEPSIS-2 criteria [20,22].

Finally, the study by Huang et al. (2023) compared two groups of studies based on different MDW cut-offs. In line with our results, they found that studies using an MDW cut-off greater than 20 showed better specificity than those using a cut-off less than 20, while sensitivity remained relatively unchanged [20].

Careful consideration is required when using MDW in daily clinical practice. The FDA has only approved its use in the ED, and limited data is available in other settings. MDW cannot distinguish with certainty between cases of sepsis and non-sepsis. If used as a stand-alone measure, it may be insufficient to rule out sepsis, particularly in populations with a higher pre-test probability [22]. Consequently, MDW is recommended as an additional test in combination with the indicators generally included in the standard of care (e.g., PCT, White Blood Cells, SOFA, pro-adrenomedullin, or SIRS) to improve sepsis diagnosis [22].

To date, there is no harmonized cut-off for MDW. As previously reported in the subgroup analysis, the adoption of different cut-offs by various studies may contribute to heterogeneity. Cut-offs estimated at or above 21 units were associated with slightly better specificity while maintaining good sensitivity compared to cut-off estimates below 21. Dichotomizing results as positive or negative using a given cut-off is a common method for evaluating and interpreting the diagnostic performance of biomarkers in clinical practice. Therefore, most published results reflect the optimal balance of sensitivity and specificity required in research settings. As Eisinger et al. (2025) [22] suggested, this method is useful for interpreting biomarker results in light of their continuous values and adjusting them to a clinically relevant threshold-based pretest probability. In clinical practice, as was carried out for PCT, providing multiple MDW cut-points may be helpful to aid clinicians in interpreting the results as unlikely, low, or high probability of sepsis [22].

It is important to note that a lower cut-off should be considered when collecting blood samples using K2-EDTA tubes than K3-EDTA tubes. Therefore, the appropriate cut-off should be adjusted according to the type of tube used. According to our data and those published by Huang et al. (2023) [20] and Eisinger et al. (2025) [22], using K3-EDTA tubes may slightly improve the specificity of MDW in comparison with the use of K2-EDTA tubes. This finding could improve the positive predictive value, but further studies are needed to confirm this result [22].

Several limitations should be noted. First, although we performed a subgroup analysis to identify sources of heterogeneity, some sources remain unidentified. Second, as shown by the studies reported in the literature, different cut-points for MDW have been published, and there is no consensus on a specific measure that could distinguish sepsis from non-sepsis conditions. In this context, the presence of different anticoagulants in the collected samples, such as K2-EDTA and K3-EDTA, may contribute to the variability of the MDW relative to the manufacturer’s predefined cut-off. Therefore, further investigation is required to determine the optimal cut-off values when using different anticoagulants. Third, the significant findings in the subgroup analysis may have been influenced by differences in the number of cases and sample size among studies, although a weighted analysis was employed. Several epidemiological studies have reported that the incidence of sepsis varies among different areas and, consequently, among hospitals. The clustering of a disease in a particular area or region may be associated with localized population or environmental characteristics. Thus, although sepsis-related incidence and mortality rates are difficult to study, they may vary due to differences in resident characteristics or places of residence [69]. Regional differences in comorbidities, health behaviors, diet, socioeconomic status, genetics, and environmental exposures may alter the risk of sepsis among countries [70]. Additionally, differences in data collection methods and case identification criteria may significantly influence the incidence of sepsis and severe sepsis in the general population [71]. Standardized definitions and data acquisition regarding sepsis and severe sepsis are warranted.

Finally, the number of studies conducted in the ED, and even more so in the ICU, remains limited, and additional research is warranted to increase the credibility of our findings.

## 12. Conclusions

This review provides additional evidence that MDW is a valuable tool for detecting sepsis in adult patients through a simple blood cell count analysis.

In the ED, the MDW measurement has a slightly better sensitivity than specificity and could assist clinicians in ruling out sepsis. In this setting, MDW results are available shortly after patient hospital admission, as they can be measured by the laboratory within a few minutes without additional personal intervention. Therefore, its application may be suitable for routine screening to assist clinicians in the early diagnosis of sepsis.

## Figures and Tables

**Table 1:** Summary findings.

Variables	Estimate
Studies	9
Number of diseased	1135
Number of non-diseased	17,232
Total patients	18,367
Prevalence	0.06
Sensitivity (95%CI)	0.8 (0.75–0.85)
Specificity (95%CI)	0.76 (0.66–0.83)
DOR (95%CI)	12.51 (6.85–22.87)
LR+ (95%CI)	3.28 (2.26–4.75)
LR− (95%CI)	0.26 (0.2–0.35)
FPR (95%CI)	0.24 (0.17–0.34)

DOR: Diagnostic Odds Ratio; LR: Likelihood Ratio; FPR: False-Positive Rate; 95%CI: 95% Confidence Interval.

**Table 2:** Subgroup analysis.

Variable	SEPSIS-2	SEPSIS-3	MDW < 21	MDW ≥ 21	Prevalence < 7%	Prevalence ≥ 7%	Sample Size < 1320	Sample Size ≥ 1320	K2-EDTA	K3-EDTA
**Number of studies**	5	4	5	4	4	5	4	5	4	4
**Sensitivity (95%CI)**	0.81 (0.74–0.87)	0.79 (0.71–0.85)	0.8 (0.73–0.85)	0.81 (0.73–0.87)	0.84 (0.78–0.89)	0.77 (0.71–0.82)	0.81 (0.73–0.87)	0.8 (0.73–0.85)	0.81 (0.74–0.87)	0.81 (0.73–0.87)
**Specificity (95%CI)**	0.83 (0.76–0.88)	0.64 (0.52–0.74)	0.66 (0.56–0.75)	0.85 (0.77–0.9)	0.81 (0.69–0.89)	0.70 (0.57–0.82)	0.7 (0.54–0.82)	0.8 (0.69–0.88)	0.66 (0.53–0.76)	0.85 (0.77–0.9)
**DOR (95%CI)**	20.8 (10.87–39.85)	6.6 (3.29–13.31)	7.62 (4.01–14.47)	23.4 (11.34–48.3)	23.0 (10.7–49.2)	7.9 (4.1–15.1)	9.87 (4.05–24.09)	15.35 (7.06–33.35)	8.29 (3.93–17.48)	23.38 (10.97–49.87)
**LR+ (95%CI)**	4.76 (3.23–7.01)	2.17 (1.55–3.06)	2.34 (1.71–3.2)	5.28 (3.42–8.17)	4.47 (2.65–7.55)	2.59 (1.72–3.89)	2.67 (1.64–4.34)	3.92 (2.4–6.4)	2.36 (1.64–3.4)	5.28 (3.33–8.39)
**LR- (95%CI)**	0.23 (0.16–0.33)	0.33 (0.22–0.49)	0.31 (0.21–0.45)	0.23 (0.15–0.33)	0.19 (0.13–0.28)	0.33 (0.24–0.44)	0.27 (0.17–0.43)	0.26 (0.18–0.36)	0.29 (0.19–0.44)	0.23 (0.15–0.33)
**FPR (95%CI)**	0.17 (0.12–0.24)	0.36 (0.26–0.48)	0.34 (0.25–0.44)	0.15 (0.1–0.23)	0.19 (0.11–0.30)	0.3 (0.19–0.43)	0.3 (0.19–0.46)	0.2 (0.13–0.31)	0.34 (0.24–0.47)	0.15 (0.1–0.23)

MDW: Monocyte Distribution Width; K2-EDTA: dipotassium ethylenediaminetetraacetic acid; K3-EDTA: tripotassium ethylenediaminetetraacetic acid; DOR: Diagnostic Odds Ratio; LR: Likelihood Ratio; FPR: False-Positive Rate; 95%CI: 95% Confidence Interval.

**Table 3:** Meta regression.

Variable	Estimate (95%CI)	*p*
Relative sensitivity level SEPSIS-3 vs. SEPSIS-2	0.98 (0.87–1.099)	0.684
Relative specificity level SEPSIS-3 vs. SEPSIS-2	0.77 (0.63–0.93)	0.012
Global test comparison		0.042
Relative sensitivity level ≥ 21 vs. <21	1.02 (0.9–1.14)	0.80
Relative specificity level ≥ 21 vs. <21	1.28 (1.09–1.51)	0.009
Global test comparison		0.033
Relative sensitivity level ≥ 7% vs. <7%	0.91 (0.83–1.01)	0.084
Relative specificity level ≥ 7% vs. <7%	0.87 (0.70–1.07)	0.191
Global test comparison		0.115
Relative sensitivity level ≥ 1320 vs. <1320	0.98 (0.87–1.1)	0.759
Relative specificity level ≥ 1320 vs. <1320	1.14 (0.91–1.44)	0.249
Global test comparison		0.436
Relative sensitivity level K3 vs. K2	1 (0.89–1.12)	0.933
Relative specificity level K3 vs. K2	1.29 (1.06–1.56)	0.017
Global test comparison		0.049

95%CI: 95% Confidence Interval; *p*: *p*-value.

## Supplementary Materials

The following supporting information can be downloaded at: https://www.mdpi.com/article/10.3390/ijms26157444/s1.
