# Supplementary material for: Monocyte Distribution Width for Sepsis Diagnosis in the Emergency Department and Intensive Care Unit: A Systematic Review and Meta-Analysis
Source: Int J Mol Sci. 2025 Aug 1;26(15):7444. doi: 10.3390/ijms26157444 (PMC12347237; doi:10.3390/ijms26157444)
Supplement: Supplementary file 1 [file ijms-26-07444-s001.zip › Table S1.pdf]

Table S1. Search strategies for selecting studies.

Database	Keywords
**Pubmed**	("ICU" OR "Intensive Care Unit" OR "ICU patients" OR "Emergency Department" OR "ED" OR "ED patients" OR "Emergency Department patients" OR "hospitalized patients" OR "patients") AND ("Monocyte Distribution Width" OR "MDW") AND ("sepsis diagnosis" OR "sepsis" OR "sepsis patients" OR "septic patients" OR "sepsis shock" OR "septic shock") NOT ("review" [pt] OR "systematic review" [pt] OR "meta-analysis" [pt])
**Scopus**	(ICU OR "Intensive Care Unit" OR "ICU patients" OR "Emergency Department" OR ed OR "ED patients" OR "Emergency Department patients" OR "hospitalized patients" OR patients) AND ("Monocyte Distribution Width" OR mdw) AND ("sepsis diagnosis" OR sepsis OR "sepsis patients" OR "septic patients" OR "sepsis shock" OR "septic shock") AND NOT (review OR "systematic review" OR "meta-analysis")
**Ovid**	((ICU or "Intensive Care Unit" or "ICU patients" or emergency or "Emergency Department" or ED or "ED patients" or "Emergency Department patients" or "hospitalized patients" or patients) and ("monocyte distribution width" or MDW) and ("sepsis diagnosis" or sepsis or "sepsis patients" or "septic patients" or "sepsis shock" or "septic shock")) not (review or "systematic review" or "meta-analysis")

**Inclusion criteria**	**Exclusion criteria**
Randomized controlled trials	Duplicates
Observational studies: cohort study, case control study, cross-sectional study	Wrong publication type: abstracts, posters, case reports, editorials, commentaries, reviews, meta-analysis, protocols for reviews, guidelines
Prospective studies	Wrong study design: survey, in vitro studies, in vivo studies, studies without a sepsis group clearly identified, studies enrolling non-consecutive patients
Retrospective studies	Out of topic
English articles	Foreign language
Adult patients admitted in ED or ICU	Inappropriate population: non-adult patients (<18y), patients admitted in other wards, pregnancy, COVID-19 patients
	Different outcomes or incomplete data
