# Supplementary material for: Monocyte Distribution Width for Sepsis Diagnosis in the Emergency Department and Intensive Care Unit: A Systematic Review and Meta-Analysis
Source: Int J Mol Sci. 2025 Aug 1;26(15):7444. doi: 10.3390/ijms26157444 (PMC12347237; doi:10.3390/ijms26157444)
Supplement: Supplementary file 1 [file ijms-26-07444-s001.zip › Table S5.pdf]

Table S5. Bivariate results to estimate heterogeneity.

Variable	Estimate
Variance of logit sensitivity	0.137
Variance of logit specificity	0.508
MOR sensitivity	1.423
MOR specificity	1.974
Bivariate I^2	0.916
Area 95% Prediction Ellipse	0.236

MOR: Median Odds Ratio.
