# Supplementary material for: Monocyte Distribution Width for Sepsis Diagnosis in the Emergency Department and Intensive Care Unit: A Systematic Review and Meta-Analysis
Source: Int J Mol Sci. 2025 Aug 1;26(15):7444. doi: 10.3390/ijms26157444 (PMC12347237; doi:10.3390/ijms26157444)
Supplement: Supplementary file 1 [file ijms-26-07444-s001.zip › Table S4.pdf]

Table S4. Characteristics of studies carried out in the Intensive Care Unit

**Authors**	**Cut-off**	**Diagnostic criteria**	**Sample size**	**Males**	**Mean age**	**Sepsis**		**Control**		**True positive**	**False positive**	**True negative**	**False negative**
						**Number**	**Mean MDW (SD)**	**Number**	**MeanMDW (SD)**				
Polilli et al. (2021) [14]	20	SEPSIS-3	180	107	63.7 ± 16.5	72	28.10 (8.16)	53	21.30 (2.71)	53	6	45	17
Agnello et al. (2021) [49]	N/A	SEPSIS-3	96	57	N/A	46	32.54 (8.69)	50	21.38 (3.47)	N/A	N/A	N/A	N/A
Piva et al. (2021) [48]	24.65	SEPSIS-3	506	346	62.78 (18-90)	108	29.71 (6.09)	394	22.05 (3.96)	71	88	308	34

MDW: Monocyte Distribution Width; SD: Standard Deviation.
