# Supplementary material for: Monocyte Distribution Width for Sepsis Diagnosis in the Emergency Department and Intensive Care Unit: A Systematic Review and Meta-Analysis
Source: Int J Mol Sci. 2025 Aug 1;26(15):7444. doi: 10.3390/ijms26157444 (PMC12347237; doi:10.3390/ijms26157444)
Supplement: Supplementary file 1 [file ijms-26-07444-s001.zip › Table S3.pdf]

Table S3. Mean differences for different subgroups

Subgroup	Number of studies	Total sepsis group	Total control group	Weight	Mean difference (95%CI)	Tau^2	Heterogeneity (I^2)	Subgroup difference p-value
Diagnostic criteria								
SEPSIS-2	5	569	7368	55.1%	6.43 (3.62-9.25)	5.53	95.2%	0.08
SEPSIS-3	4	566	9854	44.9%	4.62 (3.68-5.57)	0.14	36.5%	
Prevalence								
≥7	5	756	5756	56.8%	4.51 (3.12-5.90)	1.37	86.7%	0.01
<7	4	379	11476	43.2%	7.03 (4.13-9.94)	4.13	91.8%	
Sample size								
Number of patients ≥1320	5	782	14946	59.2%	5.48 (3.03-7.93)	2.85	94.3%	0.8
Number of patients <1320	4	353	2286	40.8%	5.79 (2.51-9.08)	4.03	87.6%	
Cut-off								
Cut-point ≥21	4	552	6607	44.8%	6.91 (3.96-9.85)	2.70	91.1%	0.02
Cut-point <21	5	583	10625	55.2%	4.46 (3.00-5.92)	0.98	79.5%	
Anticoagulant								
K3-EDTA	4	552	6607	49.4%	6.91(4.02-6.47)	2.70	91.1%	0.03
K2-EDTA	4	529	10277	50.6%	4.40 (2.34-6.47)	1.11	84.2%	

K2-EDTA: dipotassium ethylenediaminetetraacetic acid; K3-EDTA: tripotassium ethylenediaminetetraacetic acid; 95%CI: 95% Confidence Interval.
