# Supplementary material for: Monocyte Distribution Width for Sepsis Diagnosis in the Emergency Department and Intensive Care Unit: A Systematic Review and Meta-Analysis
Source: Int J Mol Sci. 2025 Aug 1;26(15):7444. doi: 10.3390/ijms26157444 (PMC12347237; doi:10.3390/ijms26157444)
Supplement: Supplementary file 1 [file ijms-26-07444-s001.zip › Table S2.pdf]

Table S2. Characteristics of studies carried out in the Emergency Department

Authors	Country	Setting	Cut-off	Diagnostic criteria	Sample size	Prevalence (%)	Male gender	Mean age	Sepsis		Control	
									Number	Mean MDW (SD)	Number	Mean MDW (SD)
Crouser et al. (2017) [51]	Ohio	ED	20.5	SEPSIS-2	1320	7.42	750	S: 50 (18-84) C: 46 (18-90)	98	22.63 (3.66)	1222	19.48 (2.55)
Poz et al. (2022) [52]	Italy	ED	20.1	SEPSIS-2	985	6.40	461	N/A	63	25.5 (6.35)	922	19.26 (3.59)
Agnello et al. (2021) [15]	Italy	ED	23.1	SEPSIS-2	703	6.83	298	S: 61.5 ± 22.3* C: 59.4 ± 21.5*	48	28.39 (6.34)*	655	19.84 (2.81)
Agnello et al. (2020) [53]	Italy	ED	23.5	SEPSIS-2	2215	3.97	1084	S: 59.5 ± 24.6* C: 55.3 ± 23.7*	88	28.31 (5.2)	2127	19.71 (3.1)
Polilli et al. (2022) [54]	Italy	ED	22	SEPSIS-2	2724	9.99	1410	S: 71.5 ± 18.5; C: 62.0 ± 20.6	272	25.4 (6.21)	2452	19.49 (3.45)
Hausfater et al. (2021) [50]	France and Spain	ED	21.5	SEPSIS-3	1517	9.49	837	61 ± 19	144	25.52 (5.06)	1373	20.56 (3.68)
Singla et al. (2022) [55]	India	ED	29	SEPSIS-2, SEPSIS-3	148	47.30	95	48.73 ± 16.17	70	28.16 (9.04)	78	21.97 (5.45)
Malinovska et al. (2022) [56]	Baltimore	ED	20	SEPSIS-3	7952	2.26	3756	48.9 ± 21.5*	180	23.94 (4.9)	7772	19 (2.78)
Woo et al. (2021) [57]	Korea	ED	19.8	SEPSIS-3	549	34.24	302	59.2 ± 13.3	188	24.21 (5.23)*	361	20.43 (4.97)
Li et al. (2022) [58]	Taiwan	ED patients with symptoms suggesting potential sepsis	20	SEPSIS-3	402	13.43	201	63.7 ± 18.9	54	26.8 (7)	348	21.94 (4.68)

ED: Emergency Department; IQR: Interquartile Range; S: sepsis group; C: control group. *Value estimated by using median, interquartile range and sample size.
